# Binding of RNA Aptamers to Membrane Lipid Rafts: Implications for Exosomal miRNAs Transfer from Cancer to Immune Cells

**DOI:** 10.3390/ijms21228503

**Published:** 2020-11-12

**Authors:** Teresa Janas, Pawel Janas, Karolina Sapoń, Tadeusz Janas

**Affiliations:** 1 Institute of Biology, University of Opole, Kominka 6, 45-032 Opole, Poland; teresa.janas@uni.opole.pl (T.J.); karolina.sapon@uni.opole.pl (K.S.); 2 Kellogg School of Management, Northwestern University, Evanston, IL 60208, USA; pawel.janas8@gmail.com

**Keywords:** exosomes, immune cells, liposomes, miRNAs, rafts, RNA aptamers, SELEX

## Abstract

Intraluminal vesicles (ILVs) are released into the extracellular space as exosomes after the fusion of multivesicular bodies (MVBs) with the plasma membrane. miRNAs are delivered to the raft-like region of MVB by RNA-binding proteins (RBPs). RNA loading into exosomes can be either through direct interaction between RNA and the raft-like region of the MVB membrane, or through interaction between an RBP–RNA complex with this raft-like region. Selection of RNA aptamers that bind to lipid raft region of liposomal membranes was performed using the selection-amplification (SELEX) method. The pool of RNA aptamers was isolated, and the binding of this pool to lipid-raft regions was demonstrated. Sequencing of clones from rafted liposome-eluted RNAs showed sequences apparently of independent origin. Bioinformatics analysis revealed the most frequent raft-motifs present within these sequences. Four raft RNA motifs, one of them an EXO motif, have been identified. These motifs appear to be most frequent both in the case of raft RNA aptamers and in the case of exosomal pro-tumoral miRNAs transferred from cancer cells to macrophages, natural killer cells and dendritic cells, thus suggesting that the selection for incorporation of these miRNAs into ILVs is based on their affinity to the raft-like region of the MVB membrane.

## 1. Introduction

Exosomes are a class of extracellular vesicle (EV), 50–120 nm in diameter, originating from endosomes [1,2,3]. They are released from cells when multivesicular bodies (MVBs) containing intraluminal vesicles (ILVs) fuse with the plasma membrane. Microvesicles (also known as microparticles, shedding vesicles, or ectosomes), 0.1–1 μm in diameter, are larger EVs that are released from cells through budding out and fission of the plasma membrane. Apoptotic bodies are large EVs (0.5–2 μm in diameter) that are shed from cells during apoptosis. Non-apoptotic membrane blebs can be shed from cancer cells in the form of the largest EVs, oncosomes, 1–10 μm in diameter. Since many preparations of vesicles contain heterogeneous collections of different classes of vesicles, authors frequently define these preparations as “extracellular vesicles”. Lipid composition of the exosomal membrane resembles that of raft microdomains enriched in sphingomyelin, cholesterol, glycosphingolipids, ceramide, phospatidylserine, lyso-phosphatidylcholine, lyso-phosphatidylethanolamine and phosphatidylcholine with short saturated fatty acids (14:0, 16:0). Surface glycoconjugates, including polysialic acid, play important roles in the interaction of exosomes with cells and between exosomes [4,5]. Interestingly, in calf bovine serum, polysialylated exosomes originate mostly from immune cells [4].

Cellular membranes coexist, at physiological temperatures, as a mixture of fluid and liquid-ordered domains influenced by membrane proteins [6]. The proposed role for cholesterol in animal plasma membranes is to reduce the tendency of membrane lipids to separate into gel and fluid phases by forming an intermediate liquid-ordered phase [7]. Rafts are heterogeneous, dynamic, cholesterol and sphingolipid enriched liquid-ordered membrane nanodomains (10–200 nm) that can form microscopic domains upon clustering [8].

Structure-dependent RNA binding was demonstrated for liquid-ordered membrane lipid rafts [9]. Binding to more highly ordered gel phase membranes was found to be stronger, but much less RNA structure-dependent. RNA sequences that bind to the liquid disordered phase of a phospholipid bilayer have previously been selected and characterized [10,11,12]. A passive membrane transporter specifically directed to the amino acid tryptophan was constructed and characterized, by combining preselected RNA–lipid bilayer affinity domains with an RNA amino acid binding site [13]. Membrane RNA aptamers that bind to amyloid protein incorporated into liposomal membrane have been selected using the selection-amplification (SELEX) method [14]. Binding of bona fide cellular RNAs under physiological ionic conditions to membranes has been shown for the first time in the case of human tRNA^Sec^ [15]. This RNA with a 5-carbon hydrophobic modification was shown to bind HeLa membranes, probably favoring raft domains containing specific lipids. For a review of RNA and lipid bilayer interactions see [16,17].

RNA loading into exosomes in mammalian cells appears to be independent of ESCRT and dependent on ceramide [18]. RNAs can be delivered to the raft-like region of MVB by RNA-binding proteins (RBPs) [2,19,20,21]. It was shown that sequence-dependent miRNA association with hnRNP (heterogeneous nuclear ribonucleoprotein) A2B1 promotes their exosomal release [19]. Interestingly, hnRNP A2B1 has an affinity to the cytoplasmic membrane regions and to ceramide [19]. Therefore, this protein can likely bind to raft-like regions within the outer layer of the MVB limiting membrane. We proposed that the process of exosome loading could also be based on direct interaction of an RNA molecule with the raft-like region of the MVB limiting membrane [22]. In the case of exosomes derived from cancer cells, exosomal miRNAs are first loaded into exosomes within the cancer cell, then they are released into the extracellular space, transported, and finally they can be internalized into immune cells.

The tumor microenvironment consists of various components such as endothelial cells, cancer associated fibroblasts (CAFs), innate immune cells, adaptive immune cells (e.g., T cells), and the extracellular matrix. The innate immune cells include dendritic cells (DC), macrophages, neutrophils, myeloid-derived suppressor cells (MDSC), mesenchymal stem cells (MSCs), and natural killer cells (NK) [23]. Of these innate immune cells, macrophages are an extremely heterogeneous population, and display both pro-inflammatory and anti-inflammatory functions. One of the immune escape strategies adopted by tumor cells is to release miRNAs in exosomes to affect immune surveillance and to induce immune suppression. In macrophages these exosomal miRNAs regulate target gene expression, thereby inducing either anti-tumor or pro-tumor effector functions [24]. Overall, exosomal miRNAs can act on the different immune cell types through three main mechanisms: functional activation, functional inhibition, and functional polarization. In particular, these miRNAs can inhibit the differentiation of dendritic cells (DCs), promote the expansion of myeloid-derived suppressor cells (MDSCs), inhibit the functions of natural killer (NK) cells, induce the apoptosis of CD8+ T cells, and foster the polarization of macrophages in M2 like-tumor associated macrophages (TAMs) [25]. In addition to exosomes, microvesicles can also regulate immune cells, e.g., colorectal cancer-derived microvesicles modulate the differentiation of human monocytes to macrophages [26].

The new concept, presented in this study, is to search for membrane RNA aptamers specific for membrane lipid rafts. The RNA aptamers are selected for lipid raft region formed within the lipid bilayer during liposome formation. We also present a correlation analysis of raft motifs within both the raft RNA aptamer sequences and the sequences of exosomal pro-tumoral miRNAs transferred from cancer cells to macrophages, natural killer cells and dendritic cells.

## 2. Results

### 2.1. Selection of a Pool of RNA Aptamers to Model Membranes Containing Lipid Rafts

**Figure 1:**
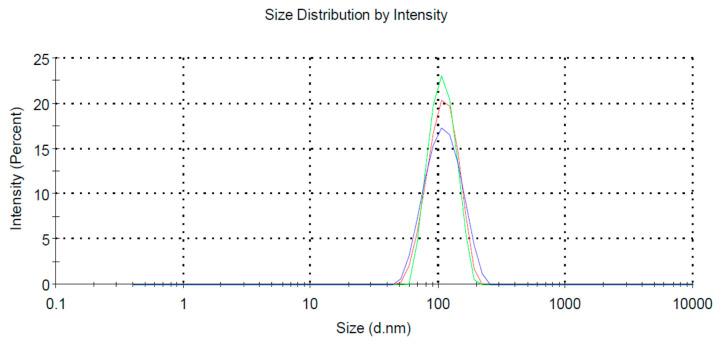
Size distribution of rafted liposomes; three profiles (blue, green and red) stand for three measurements of the same sample.

We performed the selection-amplification procedure in order to select a pool of RNA aptamers specific to lipid rafts formed within a lipid bilayer. The analysis of these rafted liposomes using a Zeta-sizer, revealed a homogenous pool of particles of ca. 100 nm in diameter (Figure 1).

**Figure 2:**
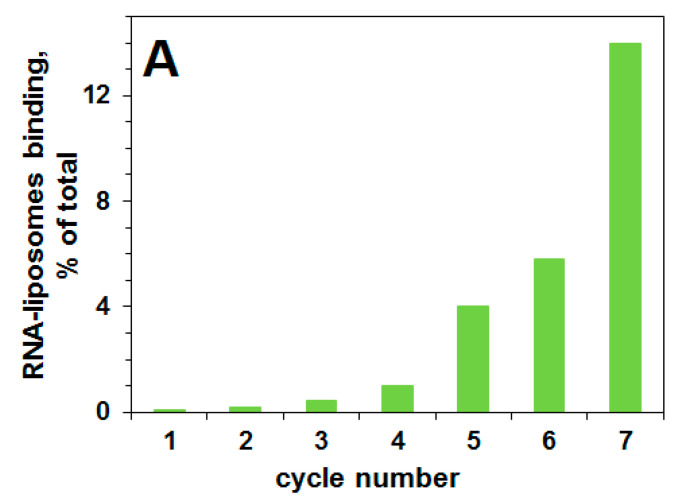

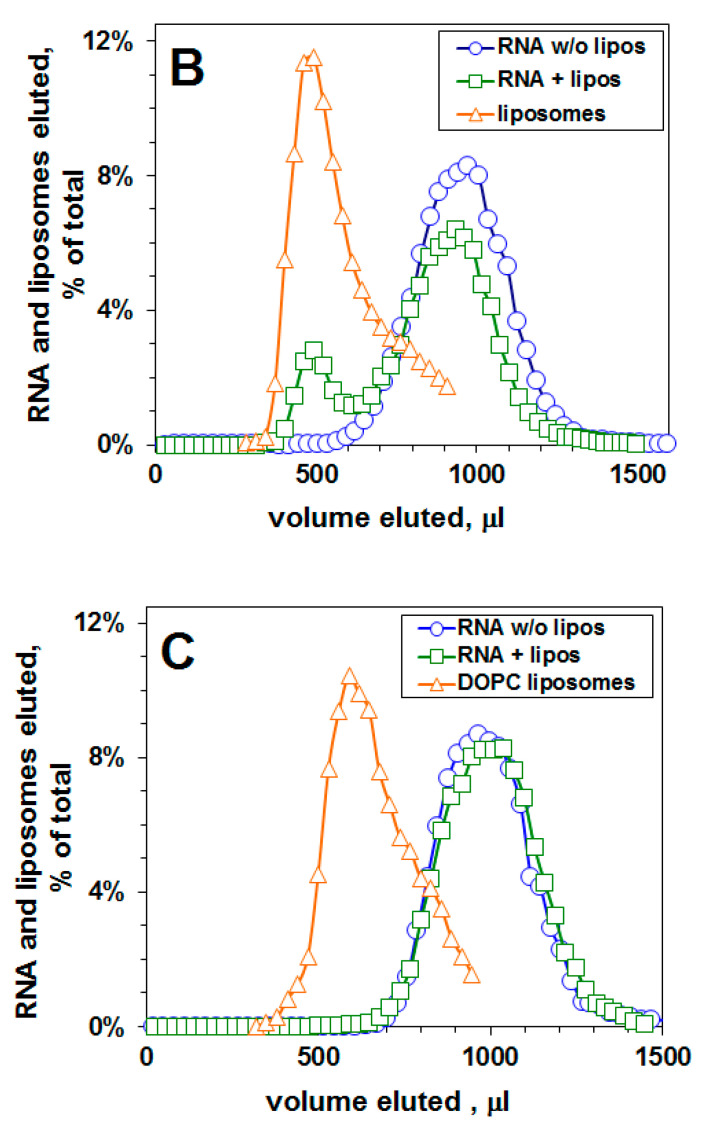
Selection of RNA aptamers specific to lipid rafts within liposomal membrane (raft-aptamers). RNA 70N/50N (100 pmol) binding to DOPC liposomes or rafted liposomes (DOPC/SM/CHOL) (0.6/0.3/0.1) in buffer 50mM KCl/HEPES, 5 mM MgCl_2_, 2 mM CaCl_2_, pH 7. (**A**)—progress of selection: percent of RNA bound to liposomes vs. cycle number. (**B**)—elution profiles of RNA and rafted liposomes after 7th cycle. (**C**)—elution profiles of RNA and DOPC liposomes after 7th cycle. The circles—elution profile of RNA without liposomes; the squares—the elution profile of RNA in the presence of liposomes; the triangles—the elution profile of liposomes (as OD_400_). RNA concentration 0.5 μM, liposome concentration 10 mg/mL.

The progress of selection for RNA aptamers that can bind liquid-ordered domains in a lipid bilayer membrane is shown in Figure 2A. The eluted fractions were analyzed for ^32^P RNA content by scintillation counting, and for liposomes by A_400_ turbidity. There is an increase in the percent of RNA bound to liposomes as a function of the cycle number. Before cycle one, there is no binding of the pool of RNA 70N/50N to the liposomal membranes containing lipid rafts.

After 7 cycles, the pool of selected RNA aptamers is tested for binding to lipid raft-containing liposomal membranes, and RNA binding is 14%. Figure 2B presents the elution profile of RNA from the Sephacryl S-1000 column after the seventh cycle. The co-elution of rafted liposomes and RNA indicates a binding of RNA molecules to the liposomal surface. There is a substantial (14%) binding of RNA to lipid-raft-containing liposomes after the seventh cycle, in contrast there is no binding of this RNA pool to control (DOPC) liposomes (Figure 2C), i.e., to liposomes without lipid rafts, thus demonstrating binding of this pool of RNA aptamers to membrane lipid rafts. Membrane raft-specific RNA aptamers were isolated from the selection cycle seven. We sequenced these clones (to obtain ca. 150 sequences) as described [27,28].

### 2.2. Binding of Raft RNA Aptamers to the Lipid Ordered Regions of Giant Lipid Vesicles (GVs)

**Figure 3:**
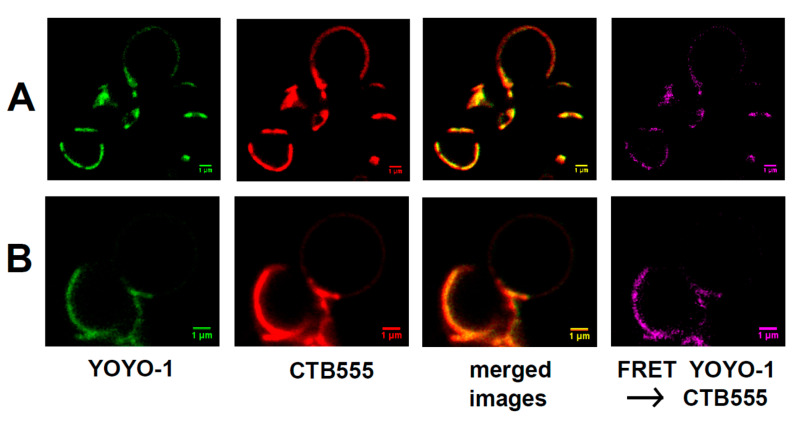
FRET microscopy for visualization of binding of raft RNA aptamers to giant lipid vesicles (GVs). Column I—YOYO-1 bound to raft RNA aptamers; column II—cholera toxin B conjugated to AlexaFluor-555 (CTB555) bound to GM1 within liquid ordered domains of giant lipid vesicle membrane (DOPC/SM/CH, 60:30:10 mole%, 0.1 mole% of ganglioside GM1); column III—merged images; column IV—FRET signal. The bars represent 1 μm. (**A**,**B**) stand for two independent experiments.

We applied FRET microscopy of giant lipid vesicles (GVs) to confirm binding of raft RNA aptamers to liquid-ordered domains (lipid raft regions) within lipid bilayers (Figure 3A,B).

In this series of micrographs (Figure 3), liquid-ordered regions were labeled with AlexaFluor555 (red) attached to cholera toxin B (CTB555) and raft RNA aptamers were labeled with YOYO-1 (green). During vesicle formation, ganglioside GM1 (0.1 mole%) was incorporated into the membrane. The fluorescence of GM1-bound toxin can be used to visualize the liquid ordered phase because GM1 molecules strongly partition into this membrane phase. The red lipid raft signal and the green YOYO-1 signal are co-localized, thus indicating that the raft RNA aptamers have coated the lipid raft domains. Lipids within the raft regions and the raft RNA aptamers are not just co-localized, but in close (nanometer range) proximity because of the strong FRET signal between CTB555 and YOYO-1.

### 2.3. Interaction of Raft RNA Aptamers to the Lipid Raft Regions of Plasma Membrane

**Figure 4:**
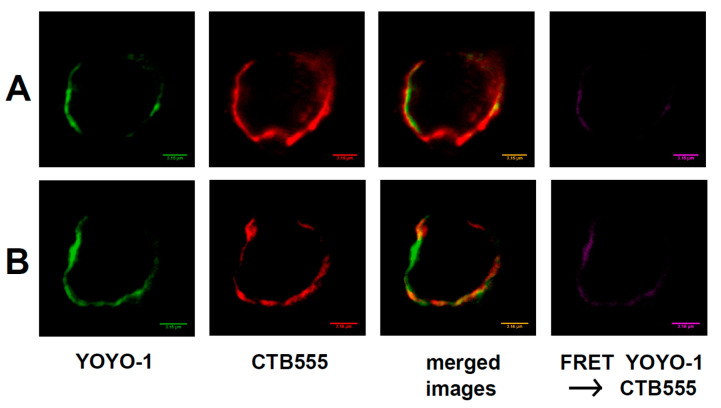
Binding of raft RNA aptamers to lipid raft regions of plasma membrane of neuroblastoma IMR-32 cells by fluorescence and FRET microscopy (representative confocal fluorescent images of IMR-32 cells): green (column I)—YOYO-1 bound to raft RNA aptamers; red (column II)—AlexaFluor-555 conjugated to cholera toxin B (CTB555) bound to the ganglioside GM1 within lipid raft region of the plasma membrane; column III—merged images; column IV—FRET signal. The bars represent 3.15 μm. (**A**,**B**) stand for two independent experiments.

Figure 4 shows the interaction of membrane-bound raft RNA aptamers with lipid raft regions of the plasma membrane of neuroblastoma cells.

We used fluorescence and FRET microscopy techniques applied to neuroblastoma cells. Raft RNA aptamers were labeled with green YOYO-1, which undergoes a large fluorescent enhancement upon intercalation into RNA structures [9], and membrane rafts were labeled with red AlexaFluor555 conjugated to cholera toxin B (CTB555) bound to the ganglioside GM1 within the lipid raft region of the plasma membrane. We observed co-localization of the signal from raft RNA aptamers and from lipid raft regions of the plasma membrane (merged images in Figure 4), and also the FRET signal (FRET YOYO-1 → CTB555 in Figure 4) from ordered lipid raft regions, showing the proximity of membrane rafts and RNA aptamers, as they would be if RNA were floating in the rafted membrane.

### 2.4. Comparison of RNA Raft Motifs with miRNA Cancer/Immune Motifs

**Figure 5:**
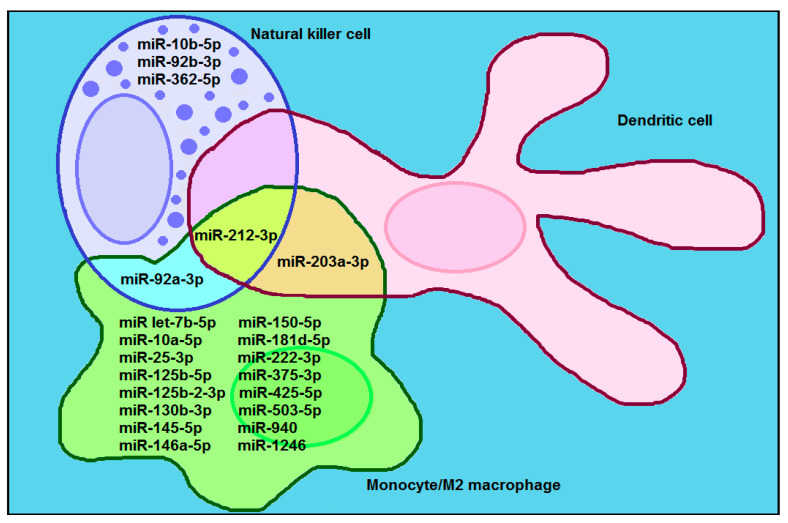
Venn diagram displaying the overlap of 17 exosomal pro-tumoral miRNAs transferred from cancer cell to macrophages, natural killer cells and dendritic cells.

We have identified, from published research (reviewed in: [23,24,25,29,30]) 17 miRNAs that are transferred within exosomes from cancer cells to innate immune cells: macrophages, natural killer cells and dendritic cells, and exert pro-tumoral function (Figure 5). Out of 17 exosomal pro-tumoral miRNAs, 11 miRNAs were exclusively transferred to monocytes/microphages, 3 miRNAs were exclusively transferred to NK cells, 1 miRNA was common between the monocytes/microphages and NK cells, 1 miRNA was common between the monocytes/microphages and dendritic cells, and 1 miRNA was common between the monocytes/microphages, NK cells and dendritic cells.

**Figure 6:**
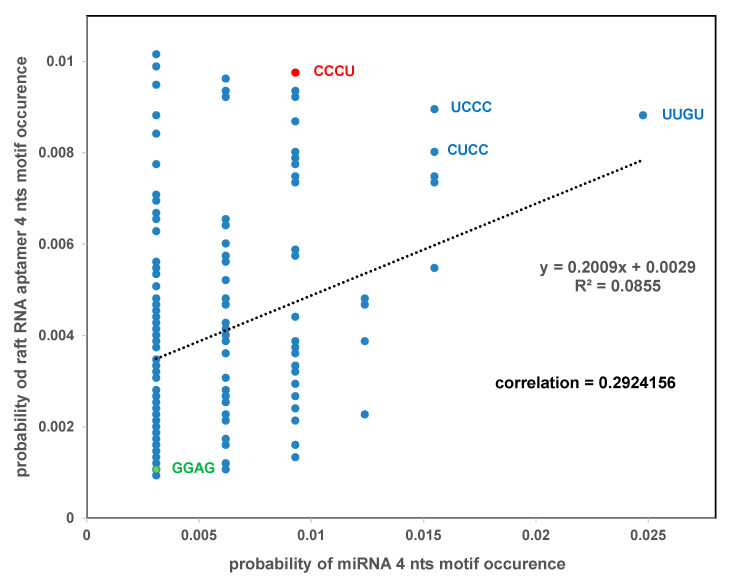
Correlation analysis of 4-nts motifs from two sets of RNAs: exosomal pro-tumoral miRNAs and raft RNA aptamers.

We performed a correlation analysis of 4-nts motifs from two sets of RNAs: exosomal pro-tumoral miRNAs (see Figure 5) and raft RNA aptamers (see Table S1). The correlation coefficient for the two sets is indicative of the strength of the relationship between these two sets. Our analysis revealed the value of the correlation coefficient equal to 0.29 thus, indicating a moderate correlation between two sets: exosomal pro-tumoral miRNAs and raft RNA aptamers. This correlation is not high because many reported “exosomal” preparations are in fact a mixture of exosomes, microvesicles and lipoproteins, and the percentage of exosomes in these mixtures is sometimes low. There are four motifs which appear to be most frequent, both in the case of exosomal pro-tumoral miRNAs and raft RNA aptamers: UUGU, UCCC, CUCC and CCCU (Figure 6).

Previously, two motifs within miRNAs were found to be significantly over-represented in exosomes from primary T lymphoblasts, and were designated as EXO motifs [19]. One of the EXO motifs, CCCU, appears to be one of the most frequent motifs both in the case of exosomal pro-tumoral miRNAs and raft RNA aptamers (see Figure 6). In contrast, the second EXO motif, GGAG, appears to be one of the least frequent motifs in both cases.

## 3. Discussion

### 3.1. RNA Aptamers Specific for Membrane Lipid Rafts

In order to show that a pool of RNA 50N/70N binds membrane lipid rafts, we used the selection-amplification (SELEX) method and the gel filtration technique on a column that voids liposomes but includes RNAs of this size. RNA aptamers are single-stranded oligonucleotides usually composed of ca. 20 to 100 nucleotides [27]. Their unique three-dimensional structures confer specificity for binding to the target. A growing number of RNA aptamers have been selected experimentally using the SELEX approach, and these aptamers have several advantages over monoclonal protein antibodies or peptides with respect to their applications in medicine. Relatively few successful selections have been reported for membrane molecular targets, in contrast to the non-membrane molecular targets [17]. To our best knowledge, the selection presented in our report, is the first selection of RNA aptamers specific to lipid membrane rafts.

RNA aptamers specific for lipid rafts (however, originating from other non-raft selections) have been identified [9], and bifunctional RNA aptamers have been constructed from an RNA membrane aptamer and an RNA aptamer specific for tryptophan [13]. These aptamers preserved their affinity to both membranes and the amino-acids. Therefore, it seems that a bifunctional RNA aptamer can be constructed from two RNA aptamers: one—specific for lipid rafts, and another—specific for a tryptophan-containing RBP. Since the lipid bilayer of the exosomal membrane is enriched in raft lipids [1], the bifunctional aptamer containing two distinct regions (first region—with affinity to lipid rafts and the second region—with affinity to RBP) can be transported (while attached to an RBP) to the lipid raft regions of the external surface of the MVB.

### 3.2. miRNA Loading into Exosomes and Exosomal miRNAs Transfer from Cancer to Immune Cells

miRNAs can be delivered to the outer (cytoplasmic) surface of the MVB limiting membrane by RBPs [2,19,20,21]. The raft regions of this membrane have a tendency for continuous inward budding, thus forming intraluminal vesicles (ILVs) [22]. At the membrane proximity, there can be three different modes of miRNA attachment to the MVB surface: I—miRNA binds directly to the membrane raft region and disconnects from the RBP; II—miRNA binds directly to the membrane raft region and does not disconnect from the RBP; III—the RBP binds to the raft region, thus miRNA is connected to the membrane surface indirectly. During ILV formation, the membrane-bound miRNA is enclosed by the ILV, and finally, the miRNAs end up inside the ILV. In the case of mode I—the miRNA is inside the ILV but is not bound to the RBP, while in modes II and III—miRNA is still bound to the RBP (Figure 4). ILVs become exosomes upon MVB fusion with the plasma membrane. The direct binding of miRNA to the raft region of the MVB limiting membrane requires a raft-binding motif within the miRNA molecule (the raft-motif) [2].

**Figure 7:**
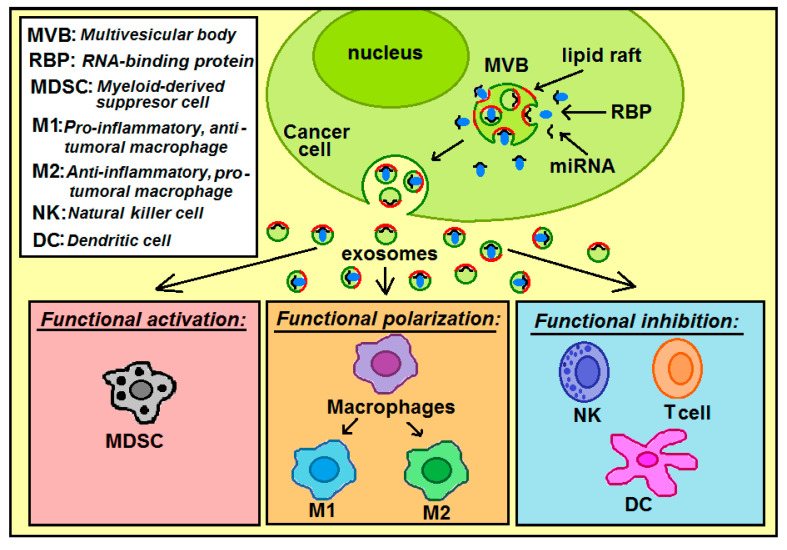
The pathway of miRNAs: from the loading process into exosomes within cancer cell, release into extracellular space, transport, and internalization into immune cells. Within the immune cells, exosomal miRNAs can act through functional activation, functional polarization or functional inhibition.

When cancer cell-derived exosomes reach the recipient immune cell, exosomal miRNAs can act on the different immune cell types through three main mechanisms (Figure 7): functional activation (MDSC cells), functional inhibition (natural killer cells, T cells, dendritic cells), and functional polarization (macrophages). Tumor-derived exosomes play critical roles in inducing the M1 or M2-like polarization of macrophages [23]. These miRNAs can induce polarization of macrophages into: anti-tumoral and pro-inflammatory M1 macrophages or pro-tumoral and anti-inflammatory M2 macrophages.

There are also other routes of tumor-derived extracellular miRNA transfer, which are independent of exosomes, but incorporate other extracellular vesicles (microvesicles, apoptotic bodies), RNA-binding proteins (RBP), or high/low-density lipoproteins (HDL/LDL) [24].

In conclusion, our selection of raft RNA aptamers and subsequent correlation analysis of 4-nts motifs from two sets of RNAs: exosomal pro-tumoral miRNAs and raft RNA aptamers, indicates that the raft-motif present within the miRNA sequence is an important factor for miRNA selective loading into cancer cell-derived exosomes.

## 4. Materials and Methods

### 4.1. Materials

Chemicals were analytical-reagent grade whenever available. Sephacryl S-1000 was obtained from Pharmacia. The following were purchased from Avanti Polar Lipids (Alabaster, AL, USA): 1,2-dioleoyl-sn-glycero-3-phosphocholine (DOPC); cholesterol (CHOL); *N*-stearoyl-d-erythro-sphingosylphosphorylcholine (Stearoyl Sphingomyelin, SM). T7 RNA polymerase (used for in vitro transcription) was obtained from Epicentre (Madison, WI, USA). Fluorescent probes: YOYO-1, cholera toxin subunit B (recombinant) Alexa Fluor 555 conjugate (CTB555) were purchased from ThermoFisher Scientific (Waltham, MA, USA). IMR-32 neuroblastoma cell line was purchased from the European Collection of Authenticated Cell Cultures (ECACC), a Culture Collection of Public Health England, UK, through Sigma-Aldrich (St. Louis, MO, USA).

### 4.2. Preparation of Large Unilamellar Vesicles (LUV)

The appropriate lipids (DOPC:SM:CHOL = 6:3:1, mole ratio) were dissolved in chloroform or chloroform/methanol (2/1), solvents were evaporated under a stream of nitrogen gas, the pellet was desiccated under vacuum for at least 2 h, resuspended in HEPES buffer (50 mM HEPES, 50 mM NaCl, 5 mM MgCl_2_ and 2 mM CaCl_2_, pH 7.0) [9]. Following a gentle vortex, the suspension was subjected to seven freeze–thaw cycles by repeated immersion in liquid nitrogen followed by warming in 60 °C water. Next, extrusion was used with a 100 nm pore diameter filter (the Avanti MiniExtruder) for LUV preparation. The mean diameter of these LUVs resembles the mean diameter of exosomes. The size distribution of liposome dispersion was determined by dynamic light scattering (DLS) by using Zetasizer Nano ZS (Malvern Instruments Ltd., Worcestershire, England) according to the manufacturer’s protocol.

### 4.3. Selection Procedure

The selection was performed as described [11]. Briefly, incubation of internally ^32^P-labeled RNA (1.0 nmol in the first selection round, decreasing in subsequent rounds) with liposomes (20 μL, 10 mg/mL) was performed in HEPES buffer at room temperature for 5 min, followed by gel filtration on a Sephacryl S-1000 1 mL column. The initial RNA pool with 50-mer random region (50N) and 70-mer random region (70N) was generated by T7 polymerase by in vitro transcription of a DNA template strand of the sequence: 5′-TGG TCA TGT GAT CGG CGT ATG—50N (or 70N)—TAT CGT GTC ATC GTC GTC CCT ATA GTG AGT CGT ATT A-3′ (the underline sequence is the T7-promoter region). Approximately 10^15^ molecules of RNA transcribed from independently synthesized DNA templates were heated in water at 65 °C for 5 min, 10xHEPES buffer was added, and solution was cooled to room temperature over 10 min to allow for RNA folding.

**Figure 8:**
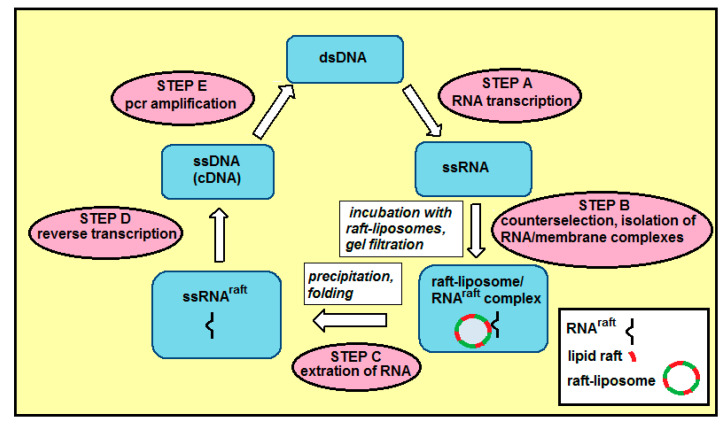
Steps of the selection-amplification (SELEX) method applied for RNA aptamers specific for membrane lipid rafts.

Selection-amplification (SELEX) procedure (Figure 8) starts with step A: T7 RNA polymerase is used to transcribe dsDNA library into a single-stranded RNA library containing ca. 10^15^ sequences. In step B, ssRNA is first incubated (counterselection) alternatively with liposomes without lipid rafts (to remove RNA sequences that bind non-specifically to liposomes) or with buffer only (to remove non-specific RNA aggregates), then ssRNA is incubated with raft-liposomes, and next the liposome/RNA complexes are separated from unbound species by gel chromatography using Sephacryl S-1000. The eluted fractions were analyzed for ^32^P RNA content by scintillation counting and for liposomes by A_320_ turbidity (Vlassov et al. 2001). Co-elution of RNA and liposomes indicates binding. RNA^raft^ are these RNA sequences that can bind to membrane lipid rafts. In step C, RNA^raft^ sequences were extracted from the liposome suspension by precipitation. In step D, the ssRNA^raft^ sequences were reverse transcribed to give a cDNA copy of the winning (i.e., enriched in the RNAs that can bind lipid rafts) sequences. In step E, pcr amplification completes the selection cycle and provides a dsDNA template enriched in the winning sequences for the next cycle.

### 4.4. Gel filtration: RNA—Liposome Binding Assay

The RNA—liposome binding assay was performed as described [9]. Briefly, ^32^P-labeled RNA in water was denatured for 3 min at 65 °C. Buffer A was added and RNA was folded by slow cooling to room temperature. LUV (5 mg of lipids/mL) were exposed to folded radiolabeled RNA (0.25 μM) for 5 min. Gel filtration of RNA/liposome mixture was performed using Sephacryl S-1000 Superfine. The eluted fractions were analyzed for ^32^P RNA content by scintillation counting and for liposomes by A_320_ turbidity. Co-elution of RNA and liposomes indicates binding. Recovery of both vesicles and RNA was typically complete (>95%).

### 4.5. Preparation of GV (Giant Vesicles)

For fluorescence microscopy, raft-containing GVs were prepared [9]. Briefly, lipids (DOPC, sphingomyelin, cholesterol, 250 nmole total) in chloroform/methanol with GM1 (0.1 mole%) were dried in a small glass vial to a thin film. After removing residual solvents (2 h in vacuum), buffer A was added. After hydration at 55 °C, the lipid thin layer was slowly cooled to room temperature. GVs (2–10 μm) were taken from the upper layer of the suspension.

### 4.6. FRET Microscopy: Binding of Raft RNA Aptamers to GVs

The fluorescence dye YOYO-1 iodide (final concentration 1.0 μM) was mixed with the pool of RNA aptamers from the seventh cycle in the HEPES buffer. There was no observable fluorescence of YOYO-1 in the presence of lipid vesicles alone [9,31]. YOYO-labeled RNA was gently mixed with GVs labeled with the fluorescent probe CTB555 at a volume ratio of 1:10, and mounted on a glass coverslip. CTB specifically interacts with GM1 residing in lipid raft domains; therefore, it is used as a lipid probe for membrane rafts [32]. Zeiss LSM 510 Meta fluorescence microscope (Carl Zeiss AG, Oberkochen, Germany) with a Plan-Apochromat 100x objective lens was used for image collection. Corrections for crosstalk for signals measured in the FRET channel were applied [9,31]. In our system, there was a 5.8% emission of YOYO-1, and 1.4% of the CTB555 excitation in the corresponding FRET channels.

### 4.7. Cell Culture

IMR-32 cells, a human neuroblastoma cell line, were cultured in Eagle’s Minimum Essential Medium (Sigma) supplemented with 1% non-essential amino acids (NEAA), 2 mM glutamine, 0.5 mg/mL of streptomycin sulfate, 100 units/mL of penicillin G, and 10% fetal bovine serum (FBS) in a 5% CO2 and 95% air humidified atmosphere at 37 °C.

### 4.8. FRET Microscopy of Neuroblastoma Cells

IMR-32 cells were grown on slides with the 8 well Thermo Scientific Nunc Lab-Tek II Chamber Slide System. After fixation with 2% paraformaldehyde for 15 min at 37 °C, and blocking (30 min room temperature) with 2% (w/v) BSA at 4 °C, the cells were incubated for 2 h at room temperature with CTB555. CTB555 was used to visualize cholesterol-enriched lipid rafts in the plasma membrane [31]. The fluorescence dye YOYO-1 iodide (final concentration 1.0 μM) was mixed with the pool of RNA aptamers from the seventh cycle, and YOYO-labeled RNA was added to the cell chamber. FRET microscopy was performed with an LSM 510 microscope (Carl Zeiss AG, Oberkochen, Germany). The signals measured in the FRET channel (sensitized FRET) were corrected for crosstalk [9,31].

## Supplementary Materials

The following are available online at https://www.mdpi.com/1422-0067/21/22/8503/s1, Table S1: 148 sequences (within the random regions) from sequencing of raft RNA aptamer clones isolated from the selection cycle 7.

## References

[1] Llorente A., Skotland T., Sylvanne T., Kauhanen D., Rog T., Orlowski A., Vattulainen I., Ekroos K., Sandvig K. (2014). Molecular lipidomics of exosomes released by PC-3 prostate cancer cells. Biochim. Biophys. Acta.

[2] Janas A.M., Sapoń K., Janas T., Stowell M.H., Janas T. (2016). Exosomes and other extracellular vesicles in neural cells and neurodegenerative diseases. Biochim. Biophys. Acta.

[3] Mathieu M., Martin-Jaular L., Lavieu G., Thery C. (2019). Specificities of secretion and uptake of exosomes and other extracellular vesicles for cell-to-cell communication. Nat. Cell Biol..

[4] Sapoń K., Gawrońska I., Janas T., Sikorski A.F., Janas T. (2020). Exosome-associated polysialic acid modulates membrane potentials, membrane thermotropic properties, and raft-dependent interactions between vesicles. FEBS Lett..

[5] Costa J. (2017). Glycoconjugates from extracellular vesicles: Structures, functions and emerging potential as cancer biomarkers. Biochim. Biophys. Acta.

[6] Hazel J.R., McKinley S.J., Gerrits M.F. (1998). Thermal acclimation of phase behavior in plasma membrane lipids of rainbow trout hepatocytes. Am. J. Physiol..

[7] McMullen T.P.W., Lewis R.N.A.H., McElhaney R.N. (2004). Cholesterol–phospholipid interactions, the liquid-ordered phase and lipid rafts in model and biological membranes. Curr. Opin. Colloid Interface Sci..

[8] Sezgin E., Levental I., Mayor S., Eggeling C. (2017). The mystery of membrane organization: Composition, regulation and roles of lipid rafts. Nat. Rev. Mol. Cell Biol..

[9] Janas T., Janas T., Yarus M. (2006). Specific RNA binding to ordered phospholipid bilayers. Nucleic Acids Res..

[10] Khvorova A., Kwak Y.G., Tamkun M., Majerfeld I., Yarus M. (1999). RNAs that bind and change the permeability of phospholipid membranes. Proc. Natl Acad. Sci. USA.

[11] Vlassov A., Khvorova A., Yarus M. (2001). Binding and disruption of phospholipid bilayers by supramolecular RNA complexes. Proc. Natl. Acad. Sci. USA.

[12] Janas T., Yarus M. (2003). Visualization of membrane RNAs. RNA.

[13] Janas T., Janas T., Yarus M. (2004). A membrane transporter for tryptophan composed of RNA. RNA.

[14] Janas T., Sapon K., Stowell M.H.B., Janas T. (2019). Selection of membrane RNA aptamers to amyloid beta peptide: Implications for exosome-based antioxidant strategies. Int. J. Mol. Sci..

[15] Janas T., Janas T., Yarus M. (2012). Human tRNASec associates with Hela membranes, cell lipid liposomes and synthetic lipid bilayers. RNA.

[16] Janas T., Janas T., Yarus M., Gesteland R.F., Cech T.R., Atkins J.F. (2005). RNA, lipids and membranes. The RNA World III.

[17] Janas T., Janas T. (2011). The selection of aptamers specific for membrane molecular targets. Cell. Mol. Biol. Lett..

[18] Kosaka N., Iguchi H., Yoshioka Y., Takeshita F., Matsuki Y., Ochiya T. (2010). Secretory mechanisms and intercellular transfer of microRNAs in living cells. J. Biol. Chem..

[19] Villarroya-Beltri C., Gutierrez-Vazquez C., Sanchez-Cabo F., Perez-Hernandez D., Vazquez J., Martin-Cofreces N., Martinez-Herrera D.J., Pascual-Montano A., Mittelbrunn M., Sanchez-Madrid F. (2013). Sumoylated hnRNPA2B1 controls the sorting of miRNAs into exosomes through binding to specific motifs. Nat. Commun..

[20] Statello L., Maugeri M., Garre E., Nawaz M., Wahlgren J., Papadimitriou A., Lundqvist C., Lindfors L., Collen A., Sunnerhagen P. (2018). Identification of RNA-binding proteins in exosomes capable of interacting with different types of RNA: RBP-facilitated transport of RNAs into exosomes. PLoS ONE.

[21] Anand S., Samuel M., Kumar S., Mathivanan S. (2019). Ticket to a bubble ride: Cargo sorting into exosomes and extracellular vesicles. Biochim. Biophys. Acta Proteins Proteom..

[22] Janas T., Janas M.M., Sapoń K., Janas T. (2015). Mechanisms of RNA loading into exosomes. FEBS Lett..

[23] Kwon Y., Kim M., Kim Y., Jung H.S., Jeoung D. (2020). Exosomal microRNAs as mediators of cellular interactions between cancer cells and macrophages. Front. Immunol..

[24] Syed S.N., Frank A.C., Raue R., Brune B. (2019). MicroRNA-A Tumor Trojan Horse for Tumor-Associated Macrophages. Cells.

[25] Raimondo S., Pucci M., Alessandro R., Fontana S. (2020). Extracellular vesicles and tumor-immune escape: Biological functions and clinical perspectives. Int. J. Mol. Sci..

[26] Baj-Krzyworzeka M., Mytar B., Szatanek R., Surmiak M., Węglarczyk K., Baran J., Siedlar M. (2016). Colorectal cancer-derived microvesicles modulate dierentiation of human monocytes to macrophages. J. Trans. Med..

[27] Ciesiolka J., Illangasekare M., Majerfeld I., Nickles T., Welch M., Yarus M., Zinnen S. (1996). Affinity selection-amplification from randomized ribo-oligonucleotide pools. Methods Enzymol..

[28] Janas T., Widmann J.J., Knight R., Yarus M. (2010). Simple, recurrent RNA binding sites for L-arginine. RNA.

[29] Graner M.W., Schnell S., Olin M.R. (2018). Tumor-derived exosomes, microRNAs, and cancer immune suppression. Semin. Immunopathol..

[30] Yi M., Xu L., Jiao Y., Luo S., Li A., Wu K. (2020). The role of cancer-derived microRNAs in cancer immune escape. J. Hematol. Oncol..

[31] Sapoń K., Janas T., Sikorski A.F., Janas T. (2019). Polysialic acid chains exhibit enhanced affinity for ordered regions of membranes. Biochim. Biophys. Acta Biomembr..

[32] Sapoń K., Maziarz D., Janas T., Sikorski A.F., Janas T. (2020). Cholera toxin subunit B for sensitive and rapid determination of exosomes by gel filtration. Membranes.

