# Supplementary material for: Binding of RNA Aptamers to Membrane Lipid Rafts: Implications for Exosomal miRNAs Transfer from Cancer to Immune Cells
Source: Int J Mol Sci. 2020 Nov 12;21(22):8503. doi: 10.3390/ijms21228503 (PMC7697632; doi:10.3390/ijms21228503)
Supplement: Supplementary file 1 [file ijms-21-08503-s001.pdf]

Table S1. 148 sequences (within the random regions) from sequencing of raft RNA aptamer clones isolated from the selection cycle 7.

35-99 UCAGUUUAUCGCCCCCCCUAAUGCU

15-145 GACAUAUAUUGGGUGCUGUCGACGUGUUUAUUAAUCGCCUUUUG

32-96 CCGUUGGCACGCUCGCAGUUGUAGUGCACGUUCAUAUUAGGGUAGC

A02-37 GCUCGUUGGACUCUAGACGCCCGUUAUCCGCCAUUCACUUCCCGCGA

23-155 UUUCAUGUGCUUUUGGGCGCCCUUGAGACAAAUUGGUCCUCUUUGACU

41-22 CUGUAGGACGUGUCUCUUCACCGUACUCACAAGAGCGGAGUGGUACAUA

54-38 UUGUGGGUUGGCGCACACCAUUCUUGCUCAUUUUCAUCUCUGGAAGUGC

56-40 UGGCGCAUUGAGCAGUAACCUGCAAUCGACUACGCUAUCCCGGGACUGG

59-43 GAUUCCAGAAUCUGCAUGCUUGUAUAACAGUUCUCCCUAACUCCCGAGC

71-55 GUAAACUCCGACUGUAAGAUAUUGCGCGAAUUUGGGUUGCCGGACACUA

01-21 UACGCGGUGUGCUGUUUCGCGUCCUAUCGACGUGCUUCGUUUCCCUCUU

04-60 UCGGAUUUCUCGCCUUUCCUUAUGCUCUCUGUAAUACGCCACUCCUGAA

07-64 UGUAGGAUGACGGUCCACCUAUUAUUCGUGCCCUAUUUCUUCCUUGCGU

09-67 UCAGUUUUUGCGCGACCCCUGGCUCUCUGGCCUUUUGUUCGACUUUGCG

12-70 GGGACAUCUGUUCUGCAAUCAAGGAAAUCUGCUCUCACAGUUCUGGGUC

23-84a CAGCAACCUCCUGAUACCACUAUUUGGCAGUAGUGGUUUCAGGCCUUUC

53-119 UUUUCCCUCAUGUAUUCGUAUUGGCCGUUGUCCCUGCUCAUCCCCUGGU

67-134 AACUGAGCUUGUCUUGUAUUCGUUUGCUUUGAGCGGCCGAUUCUAAGAG

02-99 UCAGUUUAUCGCCCCCCCUAAUGCUCCAGACAUCGUGAGGAUCAUACUA

27-159 GCUGCUAGUUGAACAGUCUUAAGCUUGAUCCCUUUCCCAAUCAACCUCC

25-1 CGAUCAGCCACAGUGUUCUGCUAUCGUCUAUCUUAUGCCCCGUAGCCAAG

26-3 UGCGCUGGCGGCCGUAUUGUUCCUCUUCCCCCUCUUGCACCCCCGUACAC

27-4 UCGCUCCGCUGAUCCCGCCGUACGCAGUGUAUCGUUCUACUUGGAUAGCA

28-5 UGUUUGACCGAGUACGCGUGACUAAGUUCUUACUGACCCGUUCCUUGAGC

29-6 CUGAUUUUCGACCGGCAUUUCUGGCCUGUGUAUUGUUCUAUCCCCCUGUU

30-7 GAUGUUCGCCUCCUUGUGUCGUGUUCUGUGAAUUCGGCUUACCUGGAUCG

31-8 ACUAGCUAAACCUGUCAUGUUCGGCCUGCUUUUUUGACUCAACUUGCCCC

32-9-TERESA-YARUS_H04 UCGGCUCGCUCUGGUCGACAACCCCGUCUUGGCCUUCUCUCCAAUAUGCU

34-11 UAUCCUCGGCUGUUUAGUGUCACUCGCCCGUCUGUAAUGCCGUCGAGAUG

35-13 AGUGCUUCUAUGGGUUUGCAGUAAGUUCGUGUCUGGACCUUUCCCUUCGU

36-14 UCAUGUAUUUGGCUCUUCGCUAUGCAUAGUAGUUACUUCCCAGACCAGAC

37-15 CGAUACGUAAUUCGCACAUAACCUGUCUUAUCCUGUUCUCGGUUGUUGGU

42-23 UCUGGCGCGCACGCAAACUUUCUUUCUCUUGCUCUCGACCACGCUGAGGA

44-25 AUGGCUAUAUAUGCGUCCUCCUUCUAGCCAUUUAUCGUAUCCUUCGAGUG

45-27 GAUUAGCUGCGAUGAGCUGCUUCGGACUCUGUGGUUAUUUGCCGGUGAUG

46-29 UUCGGUCUUUUCGCUUGUAUGAUAGUCUUGUGAUGAAGUUGCUUCCCCUU

47-30 GUGGUUCGACUUGUUCUCUCUUCCCGAUCGUCUAAUCCGGGGAUCAUAUG

48-31 UUGAAUAGGAUAGUAUCGUGACUCAUCUACUACUAGUACAACGCACUCUG

49-32 UCUGUCCACUGACUUCCUUGUUCAUGCUCAUCCCUCACUAGAAAUGUCCG

50-33 AGGUCUAUCGCAGUCUUUCUUCCCUUCAAGUCACUCUUCUAGUCCAUGGU

51-34 AGUUGUGUUGACCUAGCGUAUUAACUGGUCCGUCUUUGAUAGCGUUGCGU

52-35 CGGCUCCCCACGUCAUAGCCGUCUUUUGGGUGUUGACCUUCUUAUAGCUG

57-41 ACGCGCCACCAUCCUCGCUUUGGUUUCGUCUUACCUCUGAAGGUUCCCUG

60-44 GCCUCUGUAUAUGCACUCUAUGGUCUGAAUCUUAGCUAGCCACCCUGGGC

61-45 CGAUCUCCUUGGACAUCCUGUUUGACUGUUACAAUUCCCUAAACUCUAGC

63-47 UGCCGGACACGCGAUUACGUCGUUUGUUCACUUUGCCAUCUAAGGACUAG

65-49 UCGCUGCGUUAUUCUCCUUGCCGGGAGGCUCUUUCCCCGUUCGUCUUAUG

66-50 GCAACCGGUAUUGUAUCUGAGGCGAUUGUCCGUGGGUUGCGUUCUACUCG

67-51 ACUUGUGAUCUGUCAUUAGGAUUUGGCCUUAUGAAUUAGUGUGCCCUAAC

68-52 CGUGAGGUUUGCAUCGUCGCAGCUUUGUCUUUUACGUUUACGUUCUGGUG

70-54 CCCCUUCGCCCCUUCCUUGUUACACUAUCCUCCAUUGUACUCCCUUCCUG

72-56 AGGUGCGGCUUGGAGCGCAUGUCCAGAGCAUCCUUCGCCUAUAGGCCCUG

08-66 CUCUCCUGAUCUCAGACUAUGCGGUGAGCGCGAUCGUUCCAAAGCCUGCG

10-68 CCUCUCGACUGAAAAUACUCGUAGCUCCGGCACGUUCUGUCUCCUCUGGC

13-71 CGACCCGAUAGCUGCCCCCGAUGUGUUUAUGUCCCUGCGACCCUGCCGAC

14-73 UCUCAGGGCUAUCUGUGCUCUCGUGGUAAGUACACAGCAACGAAUUGAUG

15-74 CUCUGGUAAUCUGAUGGUCCGGCCCCUUGCCUGUGUUCUGUCUGGACGAU

16-75 AGUCGCCUCCUGGCCAAACGCUAUGUUGUACCUUUGAUGCUCAGUUGCGC

18-77 UGCAAGGUCUAGACUCAGUCAUGGAUCUAAUCUGACUUCAGCGCAAACGC

19-78 UUGCUCUAGUGAUCCACUUGCGUCUAUUGAGCGGCCUUUGCCACGGGUCA

22-83 AGUAAUAGAGAUUUACUUUUCCGUUUUAUUAGGGACAAGCGUCUUCCGGU

23-84b ACCUGUGGCGGGAUAUGUUAUCUUUGGUUCUAAAGGUUUAUUCCUGUGGU

24-86 ACAUGGUGGUGGUCACUUCCUACCUUCCCGUCUCGGAAUCGCCUCCUAUG

25-87 UGUUGAGCUUGCGCGACCUCACAUACCCUGUCAAAACACGUCUUCGUGGG

27-89 CGCCUCACUGUAGUUGGUCGCCCGGCUAGGUCCGAUUGAUCCCUUUGUCU

28-90 AACGAUUAGUGGAGUUUACCUUCUCAUUUGUCGGGUCACUUCGGCAAGUG

29-92 GCACGCUAUUACUCCCGGUAGAUGAGUUAUGCUCACUCGUUCUAAAACGC

31-94-TERESA-YARUS_G04 GCUAGGCUAACAUUGUGCUCAUGGCCGAGCGUAUGGAUCUUACGGACUUA

33-97 UGCGGAUAGUGGCGUACUAGACGCUUACCGUGAACUCCUACCCCACUGAC

36-100 UCUGCCUCGCUCACCCUGCUCAUGCCCAUAUCCUGUGCUCGACCUACCUG

37-102 CCAAUACCAUGGAUUGGUCCCUUUAUCCAGCUUCUUAACCCUUACCUGUU

38-103 GUUCGGCGUGCCAUUAUGUCCAUUGUAUGGUCGAUAUGGCCUUGCACCUC

39-104 GUGGUGCUGUGCCUCAAGGUAUGUCAGGCGCUAUGUCUUCGUACCCUAUU

41-107 UCAGAUUCUCGCCUUCAGGCACGUUGGUAAUGUAGGAAGCUUCCAAUCUG

42-108 CGAUCCGGAAUUUUGUCCACCGCCUCUCUCUUCUUAUCUGGCGUCUUAGU

43-109 ACGCGACAGGGUCUGACAGGCGUCUCCUUCCCUUUUGCAUAAUCUCCCGU

44-110 GCGAGUUGCGUAUUGCUUGCUCUUGUUCUCAUUGCCCGUCGCGCUUGGUU

46-112 UCGUGUUGUGAAUUGCUUUUAGGCCUCGCAGCGUAAACGGCUUACCCUGU

47-113 CCUUUGGCUCGGUAUUGAGUCCUUAGCCUUCUCCAUGGUUUUUUCCCUUC

48-114 UGCUUUUGCCUGUCAUGCCUACUGCACUUCGAUCAAUGCCUUCCCUCGUA

49-115 CUCCUUGUUCUGUGUACUGGUUGACUUUCGUCGGUGUCUGCGUCCGAGGG

50-116 UGCCAGACGGCGCACUCUCCAGCUAUUGACGUUGAUCAUUCGCCACGCAU

51-117 UCUACGGCUUUUGUUUCAUCGUUGCGUUCUCUGUCGACACUGAGUGCCUA

54-120 AUCCGAUGUUCCGGGAUCUCGAUUACUCUGUCAUCUCGGCAGUCCGCUGU

55-121 GUAUUCGCAUGUGUGUGAUCAGGUCAUUAUGUUUGUCCUGUGGAUGUAAC

57-123 AAAUGCAAUUCGUAGUGGUCGAGAUGAAUCGUUUGUCAAGUUACCCUCCG

58-124 ACCGGAAAUUAGUGUGCAUCCUCCUCUACGUUCAUCGAUCUUCCUUGCGU

59-125 UCUGGGUCUGUCUACUCGAUCACGUGCGCGUGGAAGUAGGUGUAUAUCAG

60-126 CCCAGGAAUUGUGUUUUGACUAGCGCCCGUUCUCAUGUUGUGUGGGUGUG

61-127 CUAUGCACUCGAUGCCGUAUGCGAACUGCUCAACUGUUUUAUUCCGCCAU

62-128 AUUCUUCCCGGCACCUACUCGUCUACAUCUUGCCAUCGGGCCCCCUUGGA

64-131 GCUCAGUUGGGUAGUAUUGUAUUGGCUGUAUCCUACCGCGUCCAGAAUGA

65-132 ACUUCUAAUAGGGUGCAUGUUAUUUGUGCUCAUACCUCAAUACCCCUGCU

66-133 UCGUAAACUACGUUGGACUGCAUUCCGAAAUAUGUCUAUAUGCGCCUACU

04-111 CUAUCAGAAUAAUGUGUUCUUUGGUUCUGUGCACACGCUGUUUCAGGGCU

05-135 CAGAUUGGGCCAUCUGUCAUAGUGAUUCUUCUUCCCAGGUAUCCACUCUA

06-136 GCAAUCAUUUGCGUCUCUUUGCUGUUGCUUAUGUGUUCGCCGCUUUAAGG

08-138 GUUCUGGAUCUCUAGCCUUUCCCCCUCUGAAUCCUCCCUUUCGCAACGCG

09-139 GCUCCGGUACUAGAAUAGUGUUCUUGUGCUUGUUUCUCUACCUCGACUCA

11-141 UCUGCGCGUCUGACGUAUCAAGAAGUAUGGACCCUGAGUAUUUAUCUGCA

12-142 UUUUGCGAACGAGCUUCAUGCCCUCUUUGAAGACUGCCUGUGUUCUCCUC

13-143 GGCCUCUGCUAAGACCACUGCGUUGCAACACAUUCCUGAUCCACCUCCAA

14-144 UAUCCGGGCGUACGAUUCGCAUGAGAGUUCCCUGUAAUUUACGGCCUGGU

16-146 AUGUGAGGAGUCGCGGUUUCUUCUGGUUUUGGUCCCAUUGGCCGAGUCAA

18-149 UCGCUCAUCACUCUCCCUACUCGUCUGUAUUCUGUCCUUGACCCCCGAUG

19-151 UCUGUCUUCUUCGCUCUGACGCGAUUUUUCCGUGCGAUUUGCGUCUUUGU

22-154 UGGUUUAGCAUCGUGGCUACUAAACCUGUCGCAUCCAUCUGCCUUCCGCG

24-156 UGCCCACAUUGACGACCUCCAGACCGUGAUGGCACCCGUGGAUACCUCGA

25-157 UGUACCAUGGCAUAUCAGCUCACAUUCUUCCUGCGCCCUCCUCCCCUGCU

26-158 GCUAUUCGGAUACGCAUCGUGUCCACCGCCGAGUCAUCGGUCGGCGCGCG

28-160 GGCGAGUGCUUUUCCUCGUCCACUGCGUUUUGUCAAAGGUUCACUGAGUC

29-161 CAGAGCUUCGGUCAUCAUCGCGCUGGGUCCAUAAAGGCCGAUAUACAGAG

30-162 UCUCGGCUUAUCGCGUUACCGUUGUGAGUAUAGCUCCUCAAACCGUCUUG

31-163 GCAUUUUAAGCGUGAAUUCUUUCGCGUUUUCGUCGGGCUAUUCCUGCCUC

33-165 UAGACUCGUCGAUUGGCCAUCGAUGGUCACUGCUCGGAGAUUGUUAGGGC

34-166 UAAACCAUAGGCACCUCGUUUGGAUGGAUACACGUUUCAGCCCCCUGAUG

35-167 AUCGCAGUUCAGUUCUCAUCGCCUUGGUCCUUAGUACUCCGACCCUGCUG

03-59 GCGCUAUUCCCCUCUGGCGUUCGUCUCAACGUCUGGCCAAGACCCGAUAUA

56-122 GGCUGUGGCUUCCGUCCUCAGGCGAUUUGUCUCGUGUUGCUUGACUAGGUUGGU

07-137 UGUUCUGUGGAUGGUCAUAUUGUAUCGUUCCCUGAUCUGUGCUCCCGUACUGCCAACUGUCCUACGCC

53-3 UCGUGCGCUUCUUCUCUCUACCUUAUCGACUCUCCUAUACCCACUCACGUCCUGUGACUUCCCACACUG

26-88 CGCUGCUGGUCCAGUUCGUAUCACUGUUCGCCUAUCCGCCGUUCUCUGACUGUUGUUUGGUUUAGCGAG

38-17 CCGUGGCACUCUUGUGUCUGUUGUGCCCUUGUAGUUGUUCGUCAUGUUUUCCGCCGUCACUCCCGUCGUU

39-19 CCGCCCUUGCUCGUUUCGGAUCCCUCCAUUCCUUGCUGAAGUCGUCCUAUCGCUCAUCCAUCCUCAUGUU

40-20 GCCCGUAAUGUGGGUUGGUUCUCUACGUUCAACGCUCGUUCUUACUCGUAUCCCACAAUCUAACCCUGCC

43-24 CGCACUUACUAUGGCCCGUGUGGUUGCGAACCUUCCCUUCCCCUGUGUCAUGAUGAGAUCGGUCUUCUGG

55-39 AGUGCUUCAUGUAUUCUGGCGCUAUGCUCUAUCGUGUGCUUGCUCUCCUGCGCGAUUUCCCUCCUCCCGU

58-42 GGGCCGUGCUCAUCCAACGUCGCCGAACGGGCUCGAUUGACCCCAUGCCGCCGAAACGCUAUCCUCGACU

62-46 CUGACGAGAGCUAGCCUUUUUGUCACCGAACUUGCUUCUCGGAGGUUCCCUUGAUCUGAUUUCUCCCCCU

64-48 CUUGUCAUGACCCCGUGUUUGUUAUCUCCGCUCUUUUCAUCUCAUAGUUUGUUCUUACGACUCGCUCUCU

69-53 UUGCGGCUAUCGUUUCCGUCUUUCCCGUACGUGGUUCUAUACCCCCAGGACUCAUCCUCUCCCCUGUUUC

05-61 UAGGUAUGGUUUGUGUCCCUCCCUACCUAGCUCUAUGAUGUGGCCUAUUCGUCGUAGCACGCUGUGGCUU

06-62 GCGUGUCGUGAUUCAUUCUUGUUUCCCCCCUCGUGUCGUAUCGAGGUGCGUAUCGAUCGCUUACGCCCCC

17-76 CCUGUCUGGUGUUUCGGUCUAAUUACGUGGCUGGAUUAGUUCAACCCGAGAUGUCCGAAUCCUUGGUAUG

20-79 CGUGCUACGUGUCUCAGCACGUAUCCUGUCAAGUAUCAUGAUCCGUGCCCUCAGCGUCCUCUGACAUCAC

21-82 UCGAUGCCGCCUCUUCUCGUGUUCACUUUCGUGCCGACGCAAGCAACACUUCUCUUUCGGCCCUGGUCUC

30-93 UCCGUCAAGGCGCGUUGCAAAUCCCGAGGUCUGUAUUUUCUUCGUUAGUCCUGCACAUUCUCCCUCGAAC

34-98 GUAACUGAAUUGACCGGCACUAACCGCUUCCUACGACUCUAUUAUUGCUGAUUUUUUGGCUAACCUGCAC

40-106 UCCUAACAUUGGCAGCGUUUUAGGUUCAUCGGUUCAUUAGCCUUCCCUUAGCUUCGUCGCUCACACGUUU

52-118 UUUGCCGGCCUCUGAUUAAGCUAUCCACUCACUAUACAACGCAAAUUCGCGUUGUCUGUUUUGUUCCGUU

63-129 AGAGUUGCAAAUUGACUUCAUCCGGUCGCUUUUGAACUACUUGAUCCUUUGGAUUAUGCCUAUCCCCUGU

68-10 GUGAAAGGAGUCGUACUUUCUCUUCGUGUGUGUGCCUCGUCUUCUUCUCCCGUUUGUCUCCCCUUUCGCU

01-69 GCCAUCGGCGUAUACCGGGGCAUUUCUGGCACACACUUAGAGUCAGUGCGUGCCUUGGGACACCCCACUG

10-140 UCGUUUUAUCUCCUCCUUUGAUCUUCCUCCCAAUUUGCUACUGAUCCAGCCCGUUGGCUGUCCCAGCUUG

17-148 GCAAAGUGUAUGCGGAUCUUUGCUCGCAGUCAUUCAUCAUCUCCUAUCUGGUUGUUUCGUCCUACUGUCU

20-152 UCACCUCAUCGCCCACGAAGAAUUGCGCCGAUCUCGGAUGGCUAUGGUUCGCAACGUGCGUUAACAGAAG

21-153 AGUAGCCUGUGUCUUUUAGCCCUUUAGUACUACUCAACUUGUUCUCUUGCCUUUUGUUAUCUUUCCUCCU

32-164 UCAGCCUACCGUAUUUUGUCUAGUGUUCAUACUAAUACGACUCCAGAACGGUCAUGAUCACGGGUCUUGU
